# Different Effects of Low Selenite and Selenium-Nanoparticle Supplementation on Adipose Tissue Function and Insulin Secretion in Adolescent Male Rats

**DOI:** 10.3390/nu14173571

**Published:** 2022-08-30

**Authors:** María Luisa Ojeda, Fátima Nogales, Olimpia Carreras, Eloísa Pajuelo, María del Carmen Gallego-López, Inés Romero-Herrera, Belén Begines, Jorge Moreno-Fernández, Javier Díaz-Castro, Ana Alcudia

**Affiliations:** 1 Department of Physiology, Faculty of Pharmacy, University of Seville, 41012 Seville, Spain; 2 Department of Microbiology and Parasitology, Faculty of Pharmacy, University of Seville, 41012 Seville, Spain; 3 Department of Organic and Medicinal Chemistry, Faculty of Pharmacy, University of Seville, 41012 Seville, Spain; 4 Department of Physiology, University of Granada, 18071 Granada, Spain; 5 Institute of Nutrition and Food Technology “José Mataix Verdú”, University of Granada, 18071 Granada, Spain

**Keywords:** selenite, nanoparticles, adipose tissue, insulin

## Abstract

Adolescence is a period of intense growth and endocrine changes, and obesity and insulin-resistance processes during this period have lately been rising. Selenium (Se) homeostasis is related to lipid metabolism depending on the form and dose of Se. This study tests the actions of low-dose selenite and Se nanoparticles (SeNPs) on white (WAT) and brown adipose tissue (BAT) deposition, insulin secretion, and GPx1, IRS-1 and FOXO3a expression in the WAT of adolescent rats as regards oxidative stress, adipocyte length and adipokine secretion. Four groups of male adolescent rats were treated: control (C), low selenite supplementation (S), low SeNP supplementation (NS) and moderate SeNP supplementation (NSS). Supplementation was received orally through water intake; NS and NSS rats received two- and tenfold more Se than C animals, respectively. SeNPs were obtained by reducing Se tetrachloride in the presence of ascorbic acid. For the first time in vivo, it was demonstrated that low selenite supplementation contributed to increased adipogenesis via the insulin signaling pathway and LCN2 modulation, while low SeNP administration prevented fat depots in WAT via the decrease in insulin signaling and FOXO3a autophagy in WAT, lowering inflammation. These effects were independent of GPx1 expression or activity in WAT. These findings provide data for dietary approaches to prevent obesity and/or anorexia during adolescence. These findings may be relevant to future studies looking at a nutritional approach aimed at pre-venting obesity and/or anorexia in adolescence.

## 1. Introduction

Adolescence is a period of intense development, associated with important physical, endocrine and neurodevelopmental changes, resulting in the modulation of body weight and composition [1]. Therefore, it has long-term implications for health, especially if obesity appears and/or insulin resistance (IR) occurs; both situations are currently increasing dramatically in this period [2]. Furthermore, the prevalence of eating disorders among adolescents, such as anorexia nervosa, is also increasing [3] and is associated with severe alterations in the metabolism of central and peripheral adipose tissue (AT), affecting overall health during this vulnerable phase [4].

AT can be classified into brown adipose tissue (BAT) and white adipose tissue (WAT) with different morphological and functional profiles. BAT confers the ability to produce heat through thermogenesis and WAT has the capacity to store energy. The BAT/WAT ratio indicates the homeostasis of AT and its response to energy or nutritional demands. WAT can be classified into visceral and subcutaneous [5]. A high amount of visceral WAT is specifically associated with obesity and metabolic dysregulation, promoting glucose intolerance and IR [6]. This is in part because visceral WAT has higher levels of mature adipocytes, which are also larger than in other AT [7]. Mature adipocytes act as an energy reservoir and are capable of secreting endocrine molecules that regulate metabolism, such as adipokines. During obesity, adipocyte hypertrophy promotes hypoxia and induces WAT inflammation and oxidative stress (OS), leading to low-grade chronic inflammation [8], associated with metabolic disturbances [9,10,11]. Moreover, adipokine secretion changes dramatically [12]. These peptides act as classic hormones that affect tissue and organ metabolism, contributing to a decrease in insulin sensitivity of tissues and inducing inflammation [13]. On the other hand, the absence or loss of WAT that occurs through lipodystrophy and anorexia–cachexia contributes to the development of hepatic steatosis and IR, since the circulating fat is deposited on the liver [14,15]. In addition, a distinct adipokine dysregulation profile appears during anorexia nervosa, following the anticipated pattern of low weight and WAT loss [16].

Selenium (Se) is a trace element with important antioxidant and anti-inflammatory properties mediated by different selenoproteins [17]. Selenoproteins, such as glutathione peroxidase (GPx) and thioredoxin reductases (TXNRDs), were initially recognized as antioxidants. Currently, it is established that the 25 known selenoproteins intricately regulate the functioning of the endocrine system and intracellular signaling [18]. Se also modulates preadipocyte proliferation and adipogenic differentiation, and it interferes with insulin signaling in WAT, which regulates lipolysis [19]. These effects are due in part to the antioxidant activity of GPx1, GPx3, GPx4 and TXNRDs but also to selenoproteins SELENOS and SELENOW, resident in the endoplasmic reticulum, and hepatokine SELENOP [20]. This occurs because reactive oxygen species (ROS) play a dual role in the regulation of both the differentiation and function of adipocytes [21]. Adipogenesis is accompanied by an increase in ROS generation, mainly during the differentiation and maturation of adipocytes [20,22]. Moreover, insulin signaling increases ROS production in WAT, leading to lipogenesis [23]; however, excessive ROS generation also impairs insulin sensitivity [24]. Thus, the balance activity of antioxidant selenoproteins ensures tight control of ROS generation during adipocyte differentiation (GPx1, GPx4, GPx3 and TXNRDs) and in mature adipocytes (GPx3 and SELENOP) [20,25,26,27]. However, these effects on adipogenesis depend on Se form and dose, being its safety range rather narrow [19].

Nanoparticles are characterized by high surface area, high solubility, thermal resistance, low toxicity, slow excretion rate and sustained release, which have beneficial effects on the metabolic, physiological and biological functions of animals [28]. Therefore, Se nanoparticles (SeNPs) can offer interesting chemical properties that improve the photoelectric, biological and therapeutic properties of Se [29]. SeNPs have been outlined for having some advantages over other organic and non-organic Se forms; for instance, they can be used at smaller concentrations to exert the same pharmacological effect, being more soluble and showing better bioavailability, since they can interact through covalent and non-covalent bonds and can easily indistinctly conjugate with various positively and negatively charged moieties [30,31]. Unfortunately, high doses of SeNP are often related not only to toxic effects but also to pro-oxidant properties and the ability to disrupt the cell-membrane integrity [32,33]. Currently, SeNPs are commonly used in the areas of biomedicine, cancer therapy and neurological diseases, and as anti-inflammatory, anti-apoptotic, anti-bacterial and antiviral agents [30]. Recently, they were used in the IR diabetic process [34]. In this context, Hassan et al. [29] showed that SeNPs in diabetic rats improved the expression of adipocyte peroxisome proliferator-activated receptor (PPARγ) in WAT; the expression in the liver of insulin receptor substrate-1 (IRS-1); and serum levels of IL-6. However, to the best of our knowledge, there are no studies analyzing the use of SeNPs in WAT development. Therefore, the aim of this study is to analyze the effects of low doses of oral selenite and SeNPs in WAT mass and function of adolescent rats to find if they exert different biological effects among these compounds.

## 2. Materials and Methods

### 2.1. Animals

Twenty-four adolescent male Wistar rats (Centre of Production and Animal experimentation, Vice-rector’s Office for Scientific Research, University of Seville) were used in these experiments. The rats were received at 21 days of age and housed in groups of two rats per cage with enrichment of the environment for one week, to acclimate them to housing and handling conditions. The experimental protocol was conducted over a 3-week period, beginning when the rats reached the postnatal day (PND) of 28 days of age and ending at 47 days of age. This period corresponds to adolescence in Wistar rats [35]. The animals were kept at an automatically controlled temperature (22–23 °C) and in a 12 h light–dark cycle (09:00 to 21:00).

**Figure 1:**
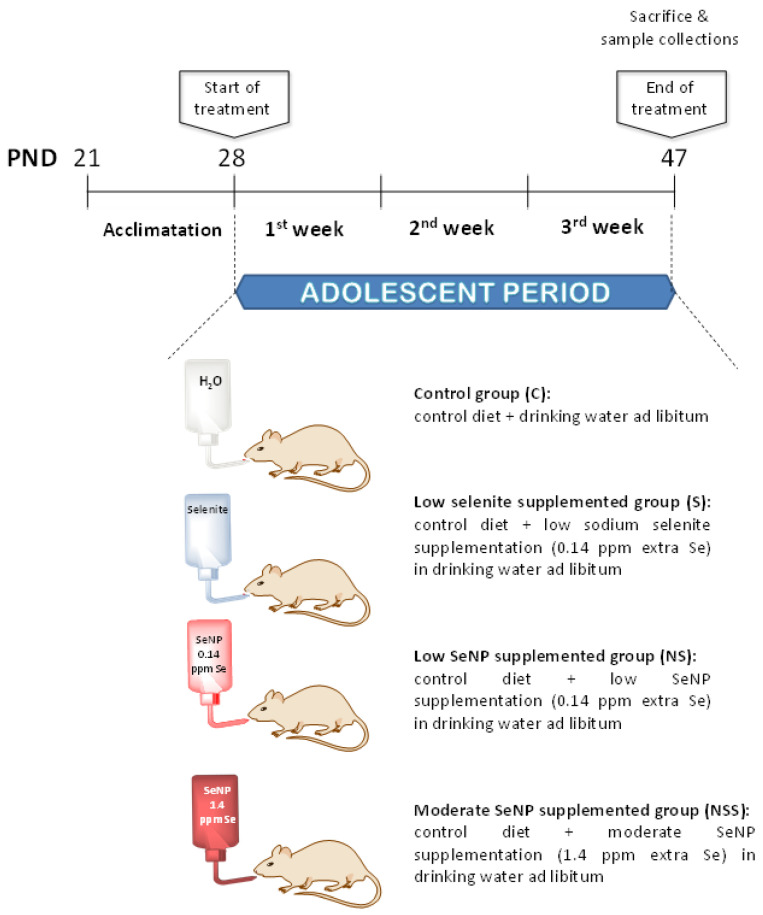
Experimental design of the study.

On PND 28, when the adolescent period began, rats with an initial weight of 49.8 ± 3.3 g were randomly assigned to four experimental groups (*n* = 6/group) according to their treatments (Figure 1): control group (C), rats received control diet and drinking water ad libitum; low-selenite-supplementation group (S), rats received control diet and low sodium selenite supplementation in drinking water ad libitum; low-SeNP-supplementation group (NS), rats were exposed to control diet and low SeNP supplementation in drinking water ad libitum; moderate-SeNP-supplementation group (NSS), rats were given the control diet and moderate SeNP supplementation in drinking water ad libitum. This latter group was studied to control the potential adverse effects of SeNP administration.

The standard pellet diet (LASQCdiet^®^ Rod14-R; Märkische, Germany) that contained 0.20 ppm of Se in the form of sodium selenite was available ad libitum in all experimental groups. Low-Se-supplementation groups (S and NS) received 0.14 ppm extra Se as anhydrous sodium selenite (Panreac, Barcelona, Spain) and SeNPs (devoloped at the Department of Organic and Medicinal Chemistry, Faculty of Pharmacy, University of Seville, Spain) in drinking water during all experimental periods. The NSS group received 1.4 ppm extra Se as SeNPs in drinking water.

Sodium selenite supplementation was estimated in order to obtain the highest GPx activity in rat plasma and liver, which, as reported by Yang et al. using sodium selenate as the Se source, was obtained with 500 μg/kg dietary Se [36]. Thus, since adolescent rats in the C group ingested about 18 g of diet/rat/day and 20 mL of water/day, a supplementation with 0.14 ppm of Se in drinking water was chosen. With this supplementation, the rats consumed about 6 µg/day of Se, which is equivalent to 500 μg/kg of dietary Se. Based on this, the amount of SeNPs was calculated to supplement adolescent rats with the same amount of Se (0.14 ppm: low supplementation) to test whether it produced a similar effect to oral selenite supplementation or not, whereas moderate SeNP supplementation (1.4 ppm Se) was tenfold higher in order to study, as described above, its potential toxic effect.

Animal-care procedures and experimental protocols were in accordance with EU regulations (Council Directive 86/609/EEC; 24 November 1986) and were approved by the Ethics Committee of University of Seville (CEEA-US2019-4).

### 2.2. SeNP Development

Chemical Se tetrachloride (SeCl_4_), ascorbic acid (C_6_H_8_O_6_), poly(sodium 4-styrenesulfonate) (PSSS) and solvents were purchased and used without further purification from Sigma-Aldrich (Madrid, Spain).

**Figure 2:**
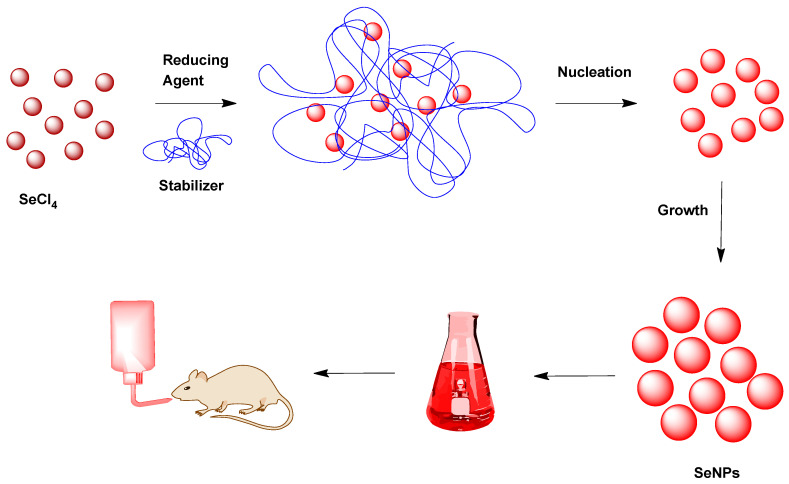
SeNP synthesis procedure via Se tetrachloride (SeCl_4_), ascorbic acid (reductor) and poly(sodium 4-styrenesulfonate) (stabilizer).

SeNPs were freshly prepared, prior to use, following the procedure previously described by Gangadoo et al. [31], in which the use of optimal quantities of ascorbic acid as a chemical reductor represents a very convenient as well as biocompatible choice to avoid other more toxic alternatives, such as sodium borohydride (Figure 2). In this method, the Se acid generated from Se tetrachloride in water was reduced with ascorbic acid. In this case, PSSS was employed to achieve better stabilization and minimize electric repulsion or aggregation, to obtain the best hydrodynamic diameter of the nanoparticles as an intense and red suspension. In a second step, the precipitation of the corresponding SeNPs was performed to obtain the portion of smaller nanoparticles (less than 50 nm). The delivery of uniform SeNPs, which were synthesized via a fast and reproducible methodology, provided a more soluble and biocompatible material that could allow us to obtain a convenient, low-toxic nutrient, characterized by higher thermal resistance and slower excretion rates, with antibacterial and antioxidant behaviors.

### 2.3. Nutritional Control

Body weight, and liquid and solid consumed by rats were monitored daily until the end of the experimental period. The amount of food and water ingested was calculated by measuring the difference between these parameters every morning and the next day using an analytical balance. Knowing the concentration of Se (ppm) in the diet and drinking water, Se intake was calculated by multiplying by the food and water ingested every day. All measurements were taken at 9:00 a.m. to avoid changes due to the circadian rhythm.

### 2.4. Samples and Anthropometric Measurements

At the end of the experimental period, the rats were fasted for 12 h using individual metabolic cages; afterwards, the adolescent rats were anesthetized with an i.p. injection of 28% *w*/*v* urethane (0.5 mL/100 g of body weight). Immediately, the cranium–caudal length (CCL) and the abdominal circumference (AC) were measured using a metric caliper, and the AC/CCL ratio was determined. The body mass index (BMI) was also calculated using the corresponding formula: body weight (g)/length^2^ (cm^2^). Blood was obtained via heart puncture and collected in tubes. Serum was prepared using low-speed centrifugation for 15 min at 1300× *g*. The abdomen was opened with a midline incision in order to obtain whole organ samples. Liver, kidneys, heart, pancreas, brain, WAT and BAT were removed, weighed, frozen in liquid nitrogen and stored at −80 °C prior to biochemical determinations. The BAT/WAT ratio was determined and the somatic index of each organ (LSI, KSI, HSI, PSI, BSI, WATSI and BATSI) was calculated by dividing the organ weight by the total animal weight.

### 2.5. Biochemical Measurements in Serum

In serum, insulin and glucose levels as well as the lipid profile (triglycerides (TG), cholesterol and HDL) were measured with an automated analyzer (Technicon RA-1000; BayerDiagnostics). VLDL and LDL serum values were estimated as follows: VLDL = TG/5; and LDL = Cholesterol − HDL − VLDL. The HDL/LDL ratio was calculated from these data.

### 2.6. Antioxidant GPx Activity and Oxidative Stress Markers in WAT

In order to measure the activity of the antioxidant enzyme GPx as well as oxidative stress markers, WAT samples from adolescent rats were homogenized (1:4 *w*/*v*) using a Potter homogenizer (Pobel 245432; Madrid, Spain) in a sucrose buffer (15 mM Tris/HCl (pH 7.4), 250 mM sucrose, 1 mM EDTA and 1 mM dithiothreitol) in an ice bath. The homogenates were centrifuged at 900× *g* for 10 min at 4 °C. Then, the resulting supernatant was employed for the biochemical assay. GPx activity (mU/mg) was determined in serum and WAT homogenates according to the technique described by Lawrence and Burk, in which GPx catalyzes the oxidation of glutathione using hydrogen peroxide and the absorbance decrease due to the oxidation of NADPH is measured at 340 nm for 3 min [37]. The oxidative stress status in WAT was evaluated via lipid and protein oxidation levels. Lipid peroxidation was determined with the colorimetric method described by Draper and Hadley, where malondialdehyde (MDA) (mol/mg protein), the end-product of the oxidative degradation of lipids, reacts with thiobarbituric acid and the final product is quantified at 535 nm [38]. Protein oxidation was measured according to the technique proposed by Reznick and Packer, in which carbonyl groups (CGs) (nmol/mg protein) are quantified at 366 nm, due to the reaction of 2,4-dinitrophenylhydrazine with CGs [39].

### 2.7. Immunoblotting Assays

WAT samples were homogenized (1:10 *w*/*v*) in 50 mM phosphate buffer (K_2_HPO_4_ 50 mM, KH_2_PO_4_ 50 mM, EDTA 0.01 mM, protease inhibitor 1:10 (Complete Protease Inhibitor Cocktail Tablets, ROCHE, Madrid, Spain)) using a Potter homogenizer (Pobel 245432; Madrid, Spain). Then, the homogenates were centrifuged at 2000 r.p.m at 4 °C for 10 min, and the final supernatant was aliquoted and frozen at −80 °C until analysis.

The expression of selenoproteins GPx1, IRS-1, FOXO3a and β-actin (as load control) in WAT homogenates was determined with the protein immunodetection technique or Western blot. The protein content of the samples was analyzed using the method by Lowry et al., and the samples for Western blot contained 25 µg of protein [40]. Proteins were separated on a polyacrylamide gel (9%) and transferred to a nitrocellulose membrane (Immobilon-P Transfer Membrane; Millipore, Billerica, MA, USA) using a blot system (Transblot; BioRad Madrid, Spain). Nonspecific membrane sites were blocked for one hour with a blocking buffer: TTBS (50 mM Tris-HCl, 150 mM NaCl, 0.1% (*v*/*v*) Tween 20; pH 7.5) and 5% milk powder (BioRad, Madrid, Spain). Then, they were probed overnight at 4 °C with specific primary antibody dilutions: GPx1 rabbit polyclonal IgG (Santa Cruz Biotechnology) (1:1000); IRS-1 rabbit polyclonal IgG (Santa Cruz Biotechnology) (1:500); FOXO3a mouse monoclonal IgG (Santa Cruz Biotechnology) (1:500); and monoclonal mouse anti β-actin IgG1A5441 (Sigma-Aldrich) (1:10,000). The next day, the probed membranes were incubated with the secondary antibody: goat Anti-Rabbit IgG (H + L) Horseradish Peroxidase Conjugate (BioRad Madrid, Spain) in dilutions of 1:3000 for GPx and 1:1500 for IRS-1 and goat Anti-Mouse IgG (H + L)-HRP Conjugate (BioRad, Madrid, Spain) in dilutions of 1:1500 for FOXO3a and 1:8000 for β-actin. Subsequently, the membranes were incubated for 1 min with a commercial developer solution, Luminol ECL reagent (GE Healthcare and Lumigen Inc., Buckinghamshire, UK), and analyzed with an Amersham Imager 600 (GE Healthcare, Buckinghamshire, UK). The quantification of the blots was performed using densitometry with the ImageJ program. The results were expressed as percent arbitrary relative units, referring to values in control animals, which were defined as 100%.

### 2.8. Adipocyte Size

To measure adipocyte size, a scanning electron microscope (SEM) operating under ultra-high vacuum (Phenom Pro desktop SEM) was used. With this microscope, information about the surface topography and composition of WAT could be recorded in 3Ds. The size of 100 adipocytes from heterogeneous areas was measured in each of the groups.

### 2.9. Adipokines

Serum adipokines such as adiponectin, resistin, adipsin, lipocalin (LCN2), plasminogen activator inhibitor-1 (PAI-1) and tumor necrosis factor (TNF)-alpha were measured using MILLIPLEX^®^ MAP Rat Adipokine Panel (Millipore Corp., St. Charles, MO, USA) based on immunoassays on the surface of fluorescent-coded beads (microspheres), following the manufacturer’s specifications (50 events per bead, 50 μL of sample, gate settings of 8000–15,000, time out of 60 s, melatonin bead set of 34). The plate was read with a LABScan 100 analyzer (Luminex Corp., Austin, TX, USA) with xPONENT software for data acquisition. The average values for each set of duplicate samples or standards were within 15% of the mean. Adipokine concentrations in plasma samples were determined by comparing the mean of duplicate samples with the standard curve for each assay.

### 2.10. Statistical Analysis

The results were expressed as means ± standard errors of the mean (SEMs), and the number of animals in each group was 6. Data from study were analyzed using statistical software (GraphPad InStat 3; San Diego, CA, USA) with the analysis of variance (one-way ANOVA). Statistical significance was established at *p* < 0.05. When ANOVA resulted in differences with values of *p* < 0.05, significant differences between means were studied with the Tukey–Kramer test.

## 3. Results

Table 1 shows that the applied low selenite and SeNP therapies enlarged the Se intake twice as much as the control and that moderate SeNP supplementation increased this intake 10 times more than in C rats. Supplemented rats (S, NS and NSS) had higher serum GPx activities than C rats; NSS rats presented higher significant values. S and NS rats ingested the same amount of food and showed a weight increase similar to that of C rats; however, NSS ones ingested less food and water, leading to a lower increase in body weight. S rats presented a longer longitude and a larger abdominal perimeter, but this increase was compensated, as the AC/CCL ratio was not affected. S rats presented higher pancreatic and BAT relative weight, being the ratio BAT/WAT augmented. NS animals had longer lengths than C ones, but they had lower BMIs and AC/CCL ratios, and lower WATSI and BAT/WAT ratios. NS rats also had a smaller abdominal perimeter, AC/CCL ratio, and a lower pancreas and BAT relative mass than S animals. Finally, NSS rats presented a lower BMI than C ones, a shorter length than S and NS rats and a higher AC/CCL than NS animals. NSS rats showed the significantly highest kidney relative weight and a higher heart mass than S rats. They also presented a lower BAT/WAT ratio than low-supplementation rats (S and NS).

**Figure 3:**
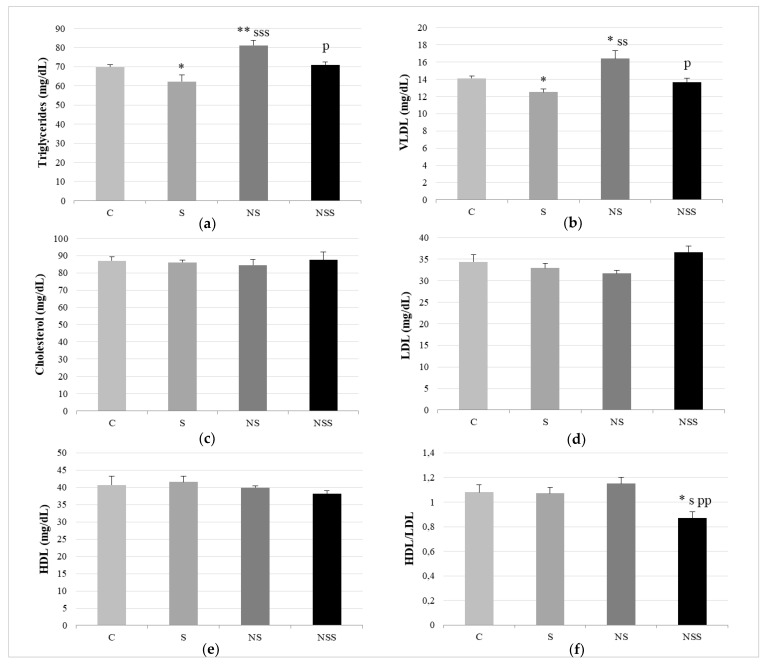
Lipid profile. Triglycerides (**a**). VLDL (**b**). Cholesterol (**c**). LDL (**d**). HDL (**e**). HDL/LDL ratio (**f**). The results were expressed as means ± SEMs and analyzed using a multifactorial one-way ANOVA followed by Tukey’s test. The number of animals in each group was 6. SEM, standard error of the mean. Groups: C, control group; S, low-selenite group; NS, low-selenite-nanoparticle group; NSS, moderate-selenite-nanoparticle group. Significance: vs. C, * *p* < 0.05, ** *p* < 0.01; vs. S, s *p* < 0.05, ss *p* < 0.01, sss *p* < 0.001; vs. NP, p *p* < 0.05, pp *p* < 0.01.

Regarding the lipid profile, S rats had lower TG and VLDL serum values than C rats (Figure 3). NS animals presented significantly higher TG and VLDL serum levels than the rest of the groups. NSS rats showed the lowest HDL/LDL ratio.

**Figure 4:**
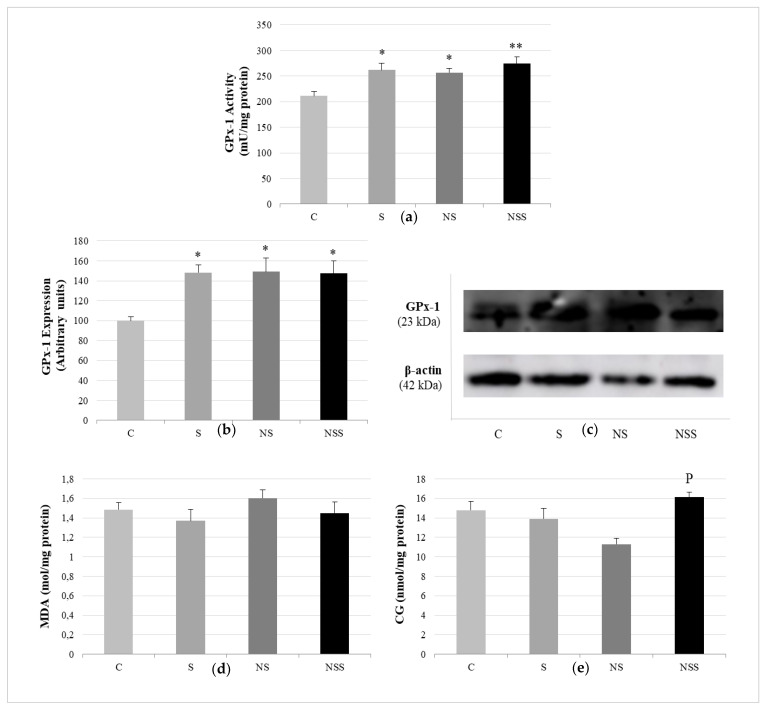
Implication of GPx1 in WAT oxidative balance. Antioxidant activity of GPx-1 (**a**). Expression of GPx-1 (expressed as percent arbitrary relative units, referring to values in control animals, which were defined as 100%) (**b**). Representative Western blot for the expression of GPx-1 and β-actin (as load control) (**c**). Lipid oxidation expressed by the levels of malondialdehyde (MDA) (**d**). Protein oxidation expressed by the levels of carbonyl groups (CGs) (**e**). The results were expressed as means ± SEMs and analyzed using a multifactorial one-way ANOVA followed by Tukey’s test. The number of animals in each group was 6. SEM: standard error of the mean. Groups: C, control group; S, low-selenite group; NS, low-selenite-nanoparticle group; NSS, moderate-selenite-nanoparticle group. Significance: vs. C, * *p* < 0.05, ** *p* < 0.01; vs. NP, p *p* < 0.05.

Figure 4 displays the data relative to oxidative balance in WAT, showing that the three supplementation groups had significantly higher GPx1 expression and GPx activity than C rats. There were no differences in these parameters among the supplementation groups; however, NSS rats presented more significant GPx activity than C ones. The oxidative lipid and protein damage was similar among the four studied groups, but NSS rats had higher levels of CGs than NS ones.

**Figure 5:**
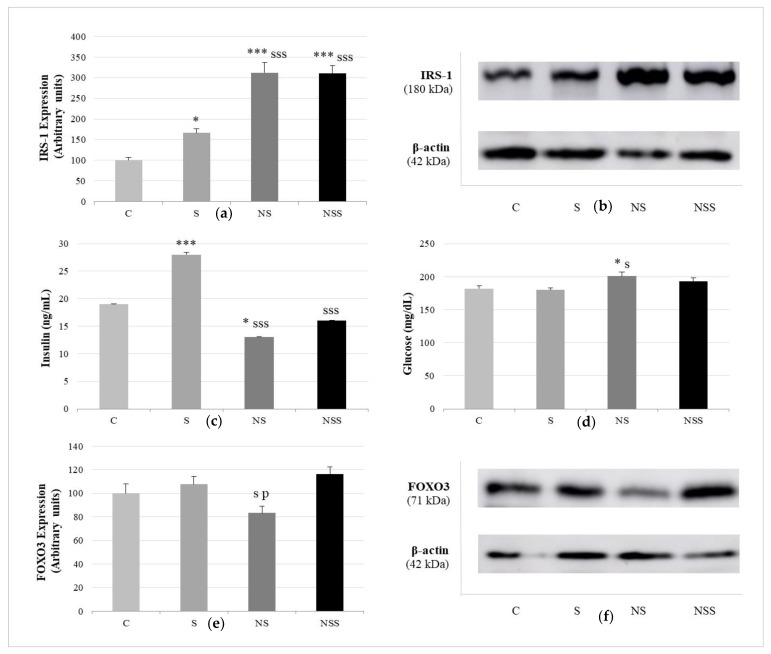
Pro-adipogenic pathways: insulin signaling and FOXO3a-autofaghy. Expression of IRS-1 (expressed as percent arbitrary relative units, referring to values in control animals, which were defined as 100%) (**a**). Representative Western blot for the expression of IRS-1 and β-actin (as load control) (**b**). Insulin levels (**c**). Glucose levels (**d**). Expression of FOXO3 (expressed as percent arbitrary relative units, referring to values in control animals, which were defined as 100%) (**e**). Representative Western blot for the expression of FOXO3 and β-actin (as load control) (**f**). The results were expressed as means ± SEMs and analyzed using a multifactorial one-way ANOVA followed by Tukey’s test. The number of animals in each group was 6. SEM: standard error of the mean. Groups: C, control group; S, low-selenite group; NS, low-selenite-nanoparticle group; NSS, moderate-selenite-nanoparticle group. Significance: vs. C, * *p* < 0.05, *** *p* < 0.001; vs. S, s *p* < 0.05, sss *p* < 0.001; vs. NP, p *p* < 0.05.

Relative to insulin function, Figure 5 shows that insulin serum levels were significantly increased in S rats vs. the rest of the groups and decreased in NS rats vs. C ones. Glucose serum values were only enlarged in NS rats with respect to C and S animals. The expression of WAT IRS-1 was increased in the three supplementation groups with respect to the C one; however, this increase was greater in NS and NSS rats, without differences among them. The marker of lipogenesis via autophagy, FOXO3a, in WAT was reduced after low exposure to SeNP compared with the expression found in S and NSS animals.

**Figure 6:**
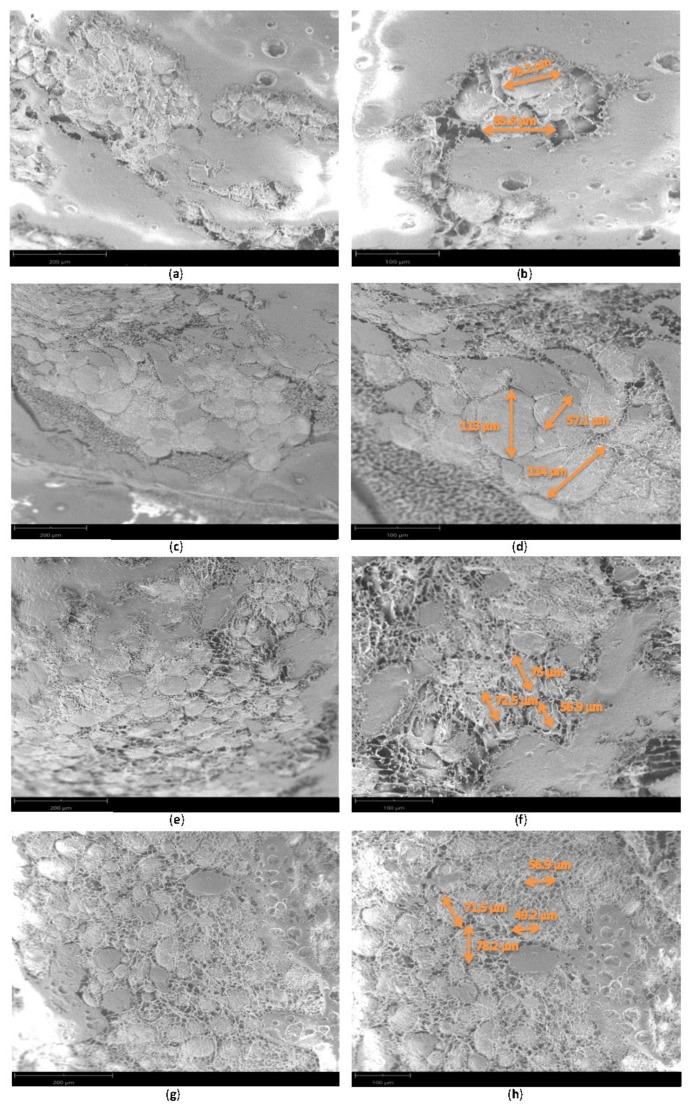
Scanning-electron-microscopy image of white adipose tissue. Control group (**a**,**b**). Selenite group (**c**,**d**). Low-selenite-nanoparticle group (**e**,**f**). Moderate-selenite-nanoparticle group (**g**,**h**).

The results obtained using a scanning electron microscope (SEM) operating under ultra-high vacuum (Phenom Pro desktop SEM) are reported in Figure 6 and showed that S rats presented the largest adipocytes (115 µm) in WAT; however, when 100 adipocytes were measured, the median data were not significantly higher than those for the control adipocytes (C, 75.4 ± 2.03 µm; S, 81.6 ± 2.03 µm). SeNP groups (NS and NSS) presented significantly lower median adipocyte size than C (*p* < 0.001) and S (*p* < 0.001) rats, which present a higher proportion of connective tissue fibers. Moreover, in SeNP-treated rats, adipocyte size was more homogeneous.

Finally, these differences in WAT tissue affected the adipokine secretion profile among groups (Table 2). S rats had the lowest adiponectin and LCN2 serum values of all experimental groups. As for NS and NSS groups, S rats also presented lower adipsin and TNF-alfa and higher t-PAI values than C animals. NS and NSS rats had higher adiponectin levels than C and S rats, especially NSS animals. Furthermore, NS and NSS animals had increased resistin and reduced TNF-alfa serum values compared with S ones.

## 4. Discussion

Low-Se-supplementation adolescent rats (S and NS) ingested the same amount of Se via the same oral route, with both forms of administration promoting bone growth and higher serum antioxidant activity of GPx. This increase in length has been previously described after the administration of selenite and SeNPs [41,42]. There is animal evidence to support the direct role of Se in bone biology not only via its promotion of osteoblastic differentiation via the inhibition of OS, ERK activation and inflammation [42,43] but also via its modulation of osteoclastogenic genes via SELENOW [44]. Moreover, Se has important endocrine functions related to growth, especially in periods of intense metabolism such as pregnancy, lactation, embryogenesis and probably adolescence, since it is an essential component of deiodinases (DIOs), regulating the contents of thyroid hormones [45,46,47]. It also regulates the transcription levels of hypothalamic and GH/IGF-axis-related genes, responsible for growth promotion [48]. Similar results have been found in different aquatic animals in which SeNP have been used for growth-promoting and feed-utilization effects [49,50,51].

However, the form of Se administration, dissolved sodium selenite or SeNPs in water, has important different impacts on abdominal circumference, a parameter associated with visceral WAT deposition [52], and increases after selenite consumption. Despite this fact, the AC/CCL ratio, a useful anthropometric correlation to detect obesity and cardiovascular and metabolic risks [53], is not affected after selenite supplementation. In accordance with this, S adolescent rats did not have larger visceral WAT depots; moreover, they presented higher BAT levels and BAT/WAT ratio than control animals, indicating that lipid homeostasis is turned towards thermogenesis, avoiding excessive lipid deposits. Therefore, S rats presented a proportionate increase in bone and lipid growth, effects that have also been detected in the progeny of dam rats supplemented with low selenite [47]. These effects of oral selenite supplementation are attributed in part to insulin up-regulation via the increase in β-cell function [17], as dietary Se is considered an insulin mimetic that, when arriving at the adipocyte, encourages adipogenesis [54]. According to that, S rats had a higher pancreatic relative mass than C rats.

In contrast, compared with selenite, SeNP supplementation did not affect pancreatic development or abdominal circumference but significantly decreased the BMI, visceral WAT depots and the AC/CCL ratio. The decrease in visceral WAT mass could be explained by the fact that SeNP are more liposoluble seleno-compounds than their anionic counterparts and easily arrive at WAT, without the need for biotransformation or specific transporters to be incorporated, unlike selenite, which is actively metabolized in cells, increasing selenoprotein synthesis [55,56]. To highlight the differences between Se nanoparticles, bearing oxidation state 0, and selenite with high oxidation state (+4), the former are known to provide slower drug delivery of Se, which would induce different bioavailability, as described by Zhang et al. [57,58]. Additionally, SeNPs may induce completely different biological interactions if compared with the ionic forms of Se to favor better adherence or larger and more reactive surfaces that catalyze binding to specific proteins [59]. Additionally, other in vivo studies have shown that SeNPs played a critical role in the up-regulation of IGF-1 gene expression and the activation of different digestive enzymes to promote and enhance intestinal-villi integrity [60,61]. The final result is that SeNPs can easily cross intestinal physiological barriers and are better absorbed in several animal models to be easily available in immunity, antioxidation and metabolism routes because of their high solubility, small size and spherical shape.

**Figure 7:**
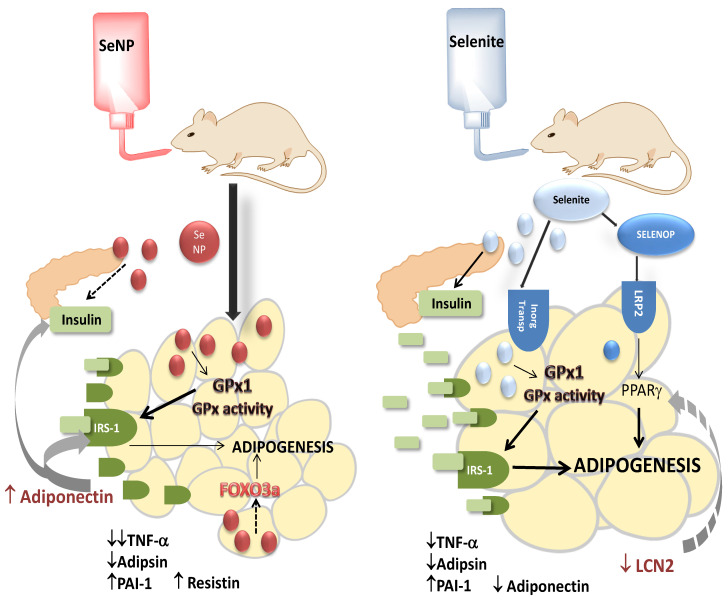
Possible mechanisms implicated in the antagonistic effects of low selenite and SeNP supplementation on WAT homeostasis during adolescence. Selenite supplementation favors adipocyte maturation and fat deposition in WAT by increasing insulin secretion and IRS-1 expression, enhancing the insulin signaling pathway and adipogenesis. It also reduces LCN2, a PPARγ inhibitor, and abrogates adipogenesis arrest. As a hypothesis, selenite could be previously transformed into SELENOP, which when arrives to LRP2 and activates PPARγ. By contrast, SeNP supplementation impairs insulin secretion, which cannot properly activate the insulin signaling pathway and adipogenesis, despite high amounts of IRS-1 being expressed and adiponectin being secreted. SeNPs also decreased FOXO3a expression, related to lipid accumulation and inflammation in adipocytes, by promoting autophagy.

Interestingly, three Se transports have been described in WAT; these pathways are: Se uptake via anion transporters (inorganic Se), methionine transporters (selenomethionine) and SELENOP-mediated transport, which uses receptors LRP2 and LRP8 to introduce SELENOP via endocytosis [62,63,64,65]. It is known that PPARγ, an important adipocyte-differentiation marker, up-regulates LRP2 expression, providing a link between adipogenesis accompanied by PPARγ and increased SELENOP uptake [66]; additionally, SELENOP plays a pivotal role during adipocyte maturation and fat deposition [20]. This route of Se transport via SELENOP could be involved during the uptake of selenite to WAT, which is less important when SeNPs are supplied, since these particles easily cross cell membranes by themselves. Therefore, a probably greater amount of direct inorganic Se in its original form arrives to adipocytes (Figure 7). According to that, in vitro studies using 3T3-L1 murine pre-adipocytes have found that direct selenite administration decreases lipid accumulation during differentiation, preventing adipogenesis. This effect could be obtained because, during physiological adipocyte differentiation and lipid accumulation, ROS signals are necessary. 3T3-L1-adipocyte exposure to selenite causes an increase in SELENOW >> GPx1 > SELENOP, leading to a significant decrease in ROS, inflammatory mediators and adipocyte-differentiation markers such as PPARγ, interfering with lipid deposition without cytotoxic effects [20]. In vivo studies using oral selenite may lead to different results.

Donma and Donma have argued that in vivo low selenite supplementation has pro-adipogenic effects via the increase in PPARγ signaling in adipocytes, which is associated with SELENOP [67] and that high selenite supply could present anti-obesity effects via the decrease PPARγ signaling; therefore, the development of lipophilic Se compounds capable of binding PPARγ could be a particular interesting approach. With the lipophilic SeNPs used in this study, this important challenge could be solved with a low dose of Se. Wang et al. have also pointed to the activation of GPx1 and SELENOP as responsible for the crossroad of the biological effect of Se in adipocytes depending on both dose and administration via the modulation of PPARγ and adipogenic differentiation in some cases and via altering the PKA/HSL pathway, which reinforces lipolysis, in other cases [68]. Since this is preliminary research, many more studies related to the SeNPs used and their adipogenic mechanisms in WAT are still necessary.

However, all data measured in this work pointed to this antagonistic effect of selenite and SeNPs on WAT homeostasis in vivo. From a lipid-profile point of view, S rats presented lower TG and VLDL serum values than C rats, avoiding ectopic lipid deposition, and NS animals had significantly higher TG and VLDL serum levels than the rest of the groups, showing that lipolysis took place. Furthermore, adipocytes from S groups were larger and presented a wider size range, indicating a correct adipogenesis process with the presence of mature adipocytes. By contrast, WAT from NS rats presented smaller fat cells, with a narrower size range and a proportionally larger amount of connective tissue fibers. Therefore, from a microscopical point of view, adipogenesis and fat depots were impaired.

SeNPs at moderate doses, although they increased serum GPx activity to a greater extent than the rest of the supplementation groups, appeared to adversely affect solid and liquid intake, leading to lower body weight and BMI and no effects on length. Furthermore, NSS adolescent rats had significantly higher relative kidney weight. These effects on solid intake and kidney development have previously been described and could suggest that toxicity occurred [30,69]. The potential toxic effects of this amount of SeNPs are primarily associated with their pro-oxidant properties and with the ability to disrupt the integrity of cell membranes [32,33]. In rats, the excess of SeNPs has been proven to induce an excessive accumulation of Se in the kidneys, which affects their correct functioning as a result of OS [70]. With respect to lipid metabolism, moderate doses of SeNPs did not affect the relative weight of WAT; nonetheless, the adipocyte size was decreased, probably in accordance with the lower weight and the appearance of dyslipidemia.

Although WAT is a tissue with relatively low Se content under physiological conditions, in the respective processes of primary preadipocytes undergoing adipocyte differentiation, ROS and selenoprotein expression in WAT drastically change, modulating adipogenesis [20,71]. An increase in H_2_O_2_ and H_2_S is needed for adipocyte differentiation, together with lower GPx1 and higher DIO2 and SELENBP1 expression. In vitro studies have found that direct selenite supplementation to 3T3-L1 preadipocytes impairs adipogenesis by increasing selenoprotein expression and decreasing ROS and inflammatory markers; however, GPx1 expression was increased in a non-dose-dependent manner [20]. Consequently, in this study, WAT GPx1 expression and activity were higher in the three supplementation groups than in control rats but without differences among them. Moreover, although ROS were not measured, indirect OS markers MDA and CGs indicated that lipids and proteins were not oxidized in WAT. GPx1 does not seem to be related to the different actions of selenite and SeNPs on WAT development. Other selenoproteins or proteins associated with Se could be involved, such as SELENOP, SELENOW or SELENBP1. Recently, interest has grown around the implication of GPx3 in obesity and IR, identifying GPx3 as a potentially novel regulator of IRS-1 expression and insulin sensitivity in WAT [72]. Insulin signaling is crucial for WAT function, since a lack of it causes adipocyte dysfunction with a marked reduction in WAT mass but not in BAT [73].

Oral bulk sodium selenite supplementation, as in a multitude of previous works [17], leads to an increase in serum insulin levels, since β cells are highly vulnerable to ROS action and their antioxidant system mainly depends not only on the main antioxidant enzymes but also on TXNRDs [74]. β-cell oxidation compromises insulin secretion. Additionally, in the pancreas, GPx1 protects from β-cell loss by inducing transcriptional factors related to proliferation and differentiation, as well as insulin production, associated with an increase in β-cell mass and insulin content [75,76,77]. A high pancreatic mass also appeared in adolescent S rats, together with normal glucose serum values and increased IRS-1 WAT expression, which supported the fact that the IR process did not appear. In addition, increased insulin secretion was related to the general anabolic process that presented in S rats. Furthermore, these effects of selenite supplementation have been used to avoid IR induced by a high-fat diet, since it alleviated IR, decreased tissue inflammation and elevated IRS-1 expression in the WAT of mice. On the contrary, low SeNPs administered to adolescent rats lead to a decrease in insulin secretion; a clear hypothesis to describe this outcome has not been found yet. To counteract the effects of hypoinsulinemia, WAT tissue expresses a much greater amount of IRS-1. However, it is not enough since the adipogenesis process is stopped and hyperglycemia further appears. According to these results, in a recent multidisciplinary study, in vivo selenite vs. in vitro selenite supplementation have been found to show different effects on WAT-insulin sensitivity during obesity and high-fat diet exposure [78]. In this case, in vitro selenite supplementation protects against IR in 3T3-L1 preadipocytes, despite in vivo studies in mice showing no selenite-induced improvement in insulin sensitivity, with only a modest effect on adipocyte morphology and enhanced insulin production in the pancreas. Once again, the way in which selenite arrives at seems to play an important role in its biological properties in this tissue. In that study, as in the study by Hauffe et al., in 3T3-L1 preadipocytes, selenite treatment via Gpx3 expression has been observed to enhance IRS-1 expression via the activation of transcription factor Sp1 [72].

FOXO3a, a Forkhead box O member of the transcription factor family, plays a critical role in a variety of biological processes. Recently, it has been described as a protein that regulates lipid accumulation and adipocyte inflammation in adipocytes through autophagy in visceral AT from obese mice and during the differentiation of 3T3-L1 adipocytes [79]. These authors have concluded that FOXO3a could promote lipid accumulation and inflammation in adipocytes by targeting autophagy. Furthermore, other authors have described that FOXO3a-dependent genes are significantly up-regulated in AT in a direct relationship with PPARγ activity [80]. With these premises, the results found in the FOXO3a expression of rats supplemented with S and NS were in consonance with the rest of the observed data. NS rats had lower expression of FOXO3a, indicating that lipid accumulation in adipocytes did not take place and inflammation was decreased, which was probably related to lower PPARγ activity and was in agreement with the lower WAT mass detected. Once again, the form of selenite administration differently affected adipocyte function.

Finally, moderate SeNP supplementation did not affect insulin secretion and glycaemia but highly increased IRS-1 in WAT; nonetheless, adipogenesis and FOXO3a seemed not to be affected. In this case, not only the form of administration but also the dose of SeNP had repercussions on WAT function in different ways.

AT is considered a dynamic endocrine organ, as it secretes a wide range of adipokines, depending on its own metabolic and energy homeostasis. Therefore, the administration of dissolved bulk selenite and SeNPs altered adipokine homeostasis in adolescent rats. In this context, independently of the dose or form of Se supplementation to adolescent rats, serum adipsin and TNF-alfa levels were decreased, and PAI-1 increased. The lower TNF-alfa values detected indicated that Se supplementation had anti-inflammatory properties, which were higher when it was supplied as SeNPs. This specific and high anti-inflammatory effect of SeNPs has been extensively described in the literature [81]. Furthermore, these results indicated that bulk selenite supplementation, despite the increased anabolism, was not related to low-grade inflammation, an important step to deliver obesity. Adipsin is known to stimulate insulin secretion from β cells and improve glucose tolerance; it also modulates WAT homeostasis and is down-regulated during obesity [82]. Because of that, it has been extensively analyzed in the IR process and pointed at as a biomarker in diabetes; it preserves β-cell mass by improving β-cell survival and maintaining their transcriptional identity [83]. In S adolescent rats, adipsin detriment did not affect insulin secretion, since bulk selenite is directly associated with an increase in β-cell mass and insulin secretion [17]; however, this decrease could be responsible, in part, for the lower insulin secretion found in NS animals in response to glucose. PAI-1 is an acute-phase protein expressed in adipocytes, but it can be highly expressed by other cells, such as hepatocytes in response to stress. Its classical role is to inhibit the plasminogen activator, blocking fibrinolysis and contributing to endothelial dysfunction. Adipocyte-derived PAI-1 is released into the circulation in parallel with increased fat mass and plays crucial roles in the insulin actions in the liver, muscle and fat. Adipocyte-derived PAI-1 influences metabolism towards TG release [84]. In addition, PAI-1 secretion is modulated by TGs in the liver, since Se is a mineral intimately related to lipid homeostasis [85,86]; perhaps, Se supplementation therapies (S, NS, NSS) and PAI-1 synthesis could be related via lipid homeostasis, which was altered in the three experimental groups.

Although these three adipokines are modulated in the same direction after Se supplementation, in this study, the form of Se administration, but not the dose, differently affected two adipokine secretions, Lipocalin-2 (LCN2) and resistin; the former was down-regulated after selenite supplementation, and the latter was up-regulated after SeNP treatments. LCN2 is also known as neutrophil gelatinase-associated lipocalin (NGAL), responsible for the transport of small and hydrophobic molecules; it plays different functions, such as antibacterial, anti-inflammatory and protection against cell stress; clinically, it is used as a biomarker for renal injury [87]. Recent reports have indicated a role for LCN2 in the modulation of insulin sensitivity, glucose and lipid metabolism. LCN2 expression is elevated by agents that promote IR and is reduced by PPARγ agonists thiazolidinediones (TZDs), an important class of insulin sensitizers used in the treatment of diabetes [88]. In this case, the selenite supplementation used in this study mimicked the activity of TZDs; this similitude has been previously reported by Wang et al. [68]. These authors have described that selenite supplementation increased adipocyte differentiation and fat deposits in AT, reducing ectopic lipid content, ROS generation and mitochondrial dysfunction in the liver and protecting against high-fat-diet-induced IR, such as TZDs [68]. Supplementation with SeNPs did not affect LCN2 secretion, but as compared with S rats, their use increased serum resistin values. Resistin is expressed in AT, but it is also found in other tissues; notably, its content in AT is proportional to the intensity of macrophage infiltration, which is the main source of this adipokine. Therefore, it has been recently described as a novel host defense peptide of innate immunity [89]. In WAT, it can stimulate lipolysis to promote the inappropriate release of fatty acids into the circulation [90], sometimes linked to IR development; both situations seemed to be stablished in SeNP-treated animals.

Finally, adiponectin secretion was very sensitive to the form and dose of Se administration, as it was down-regulated after bulk selenite supplementation and up-regulated in a dose-dependent manner after SeNP administration. This anti-inflammatory adipokine with adipogenic and insulin sensitizing effects affects fatty acid oxidation and glucose uptake in peripheral tissues, promoting appropriate lipid storage to avoid ectopic fat storage [91]. In WAT, it increases glucose uptake and adipogenesis and decreases inflammation; moreover, it specifically contributes to the increase in IRS-1 expression in WAT [92]. This promotion of WAT IRS-1 expression was clearly detected in SeNP-supplemented rats. Moreover, adiponectin also promotes beta-cell survival; it stimulates insulin secretion by enhancing the exocytosis of insulin granules and upregulating the expression of the insulin gene. In addition, it has antiapoptotic properties in β cells [91,93]. Maybe for this reason, its value was not increased in S rats, since they presented a great amount of insulin serum levels, and it was increased in NS rats, since their insulin Se levels were decreased. The higher adiponectin secretion found in NSS rats could be related to the weight loss that they suffered, since anorexia nervosa, anti-obesity medication, weight-loss diet or bariatric surgery deeply increased the overall adiponectin concentration [94].

## 5. Conclusions

For the first time, in an experimental in vivo study, it was demonstrated that the form of oral low Se administration, dissolved sodium selenite or SeNPs in water, differently affected WAT homeostasis and function. Selenite supplementation during adolescence favored adipogenesis by promoting insulin secretion and sensitivity leading to a general anabolism, without obesity or inflammation, in which the adipokine LCN2 played a pivotal role. By contrast, SeNP supplementation during adolescence impaired adipogenesis and fat deposits in WAT. These effects were due in part to lower insulin secretion, which could not properly activate the insulin signaling pathway in WAT, despite the high amount of IRS-1 that was expressed and the adiponectin amount secreted. Moreover, SeNPs have a well-known anti-inflammatory action in WAT, in part by decreasing the lipogenic and pro-inflammatory FOXO3a expression. Therefore, low selenite may be considered as a pro-adipogenic therapy, while low SeNP administration prevented adipocyte differentiation. These findings could provide important novel dietary approaches to prevent obesity and/or anorexia nervosa during adolescence, two important metabolic disturbances that are dangerously increasing in the adolescent population.

Despite a control animal group was included during the whole experimental protocol, general growth and metabolic data at baseline were not collected. For this reason, a major limitation of this study was the fact that the design was based on a single time point.

## Figures and Tables

**Table 1:** Nutritional and anthropomorphic parameters at the end of the experimental period.

Parameter	C	S	NS	NSS
Body weight increase (g/day)	6.01–0.1	6.07–0.2	5.98–0.2	5.13–0.2 * s
Solid intake (g/day)	18.26–0.35	18.58–0.18	18.28–0.09	16.54–0.19 *** sss ppp
Liquid intake (mL/day)	21.4–0.66	21.54–0.7	19.16–0.77	16.67–0.45 *** sss pp
Total Se intake (µg/day)	3.48–0.08	6.81–0.14 **	6.59–0.09 **	31.35–1.43 *** sss ppp
Serum GPx activity (mU/mg protein)	59.7–2.2	73.3–3.5 *	74.8–3.4 *	80.4–3.7 **
BMI (Kg/m^2^)	51.65–0.8	48.72–00.5	47.46–0.9 **	47.99–0.6 *
CCL (cm)	18.22–0.11	19.68–0.19 ***	19.6–0.2 ***	18.87–0.18 s p
AC (cm)	10.56–0.46	11.52–0.26 *	10.16–0.35 ss	10.75–0.25
AC/CCL	0.57–0.01	0.59–0.01	0.51–0.01 ** sss	0.57–0.01 p
LSI (%)	3.73–0.09	3.65–0.1	3.93–0.09	3.97–0.06
KSI (%)	1.04–0.04	0.99–0.03	0.98–0.02	1.19–0.03 *** ss ppp
HSI (%)	0.51–0.02	0.49–0.01	0.53–0.01	0.56–0.02 s
PSI (%)	0.72–0.02	0.89–0.03 **	0.70–0.02 ss	0.81–0.02
BSI (%)	1.08–0.02	1.17–0.02	1.07–0.02	1.14–0.03
WATSI (%)	0.62–0.02	0.59–0.03	0.50–0.02 *	0.59–0.02
BATSI (%)	0.24–0.01	0.28–0.01 *	0.25–0.02 s	0.22–0.01
BAT/WAT	0.4–0.02	0.48–0.02 *	0.49–0.02 *	0.38–0.02 s p
BMI (Kg/m^2^)	51.65–0.8	48.72–00.5	47.46–0.9 **	47.99–0.6 *

The results were expressed as means ± SEMs and analyzed using a multifactorial one-way ANOVA followed by Tukey’s test. The number of animals in each group was 6. BMI, body mass index. CCL, cranium–caudal length. AC, abdominal circumference. LSI, liver somatic index. KSI, kidney somatic index. HSI, heart somatic index. PSI, pancreas somatic index. BSI, brain somatic index. WATSI, white-adipose-tissue somatic index. BATSI, brown-adipose-tissue somatic index. BAT/WAT, brown–white adipose tissue ratio. SEM, standard error of the mean. Groups: C, control group; S, low-selenite group; NS, low-selenite-nanoparticle group; NSS, moderate-selenite-nanoparticle group. Significance: vs. C, * *p* < 0.05, ** *p* < 0.01, *** *p* < 0.001; vs. S, s *p* < 0.05, ss *p* < 0.01, sss *p* < 0.001; vs. NP, p *p* < 0.05, pp *p* < 0.01, ppp *p* < 0.001.

**Table 2:** Adipokines, hormones secreted by WAT against different stimuli, most of them metabolic and energetic.

Adipokine	C	S	NS	NSS
Adiponectin (pg/mL)	71.1–4.9	29.2–1.1 **	99.2–8.1 * sss	362–9.7 *** sss ppp
Resistin (pg/mL)	48.1–2.9	41.5–1	53.5–1.9 ss	54.1–1.9 ss
Adipsin (pg/mL)	319–12	112.7–1 ***	108–1.6 ***	112–3.5 ***
Lipocalin (LCN2) (pg/mL)	26.3–1.5	13.8–0.7 ***	29.4–1.1 sss	28.9–1.1 sss
PAI-1 (pg/mL)	70.2–1.1	90.8–1.8 ***	85.2–1.5 ***	83.4–0.7 *** s
TNF-alfa (pg/mL)	13.7–0.8	9.9–0.3 **	2.7–0.2 *** s	3.1–0.2 *** s

The results are expressed as mean ± SEM and analysed by a multifactorial one-way ANOVA followed by Tukey’s test. The number of animals in each group is 6. WAT: white adipose tissue. SEM: standard error of the mean. Groups: C, control group; S, low-selenite group; NS, low-selenite-nanoparticle group; NSS, moderate-selenite-nanoparticle group. Signification: vs. C: * *p* < 0.05, ** *p* < 0.01, *** *p* < 0.001; vs. S: s *p* < 0.05, ss *p* < 0.01, sss *p* < 0.001; vs. NP: ppp *p* < 0.001.
